# Stretchable Low Impedance Electrodes for Bioelectronic Recording from Small Peripheral Nerves

**DOI:** 10.1038/s41598-019-46967-2

**Published:** 2019-07-22

**Authors:** Francesco Decataldo, Tobias Cramer, Davide Martelli, Isacco Gualandi, Willian S. Korim, Song T. Yao, Marta Tessarolo, Mauro Murgia, Erika Scavetta, Roberto Amici, Beatrice Fraboni

**Affiliations:** 1 Department of Physics and Astronomy, University of Bologna, Bologna, Italy; 2 Department of Biomedical and Neuromotor Sciences - Physiology, University of Bologna, Bologna, Italy; 3 Department of Industrial Chemistry, University of Bologna, Bologna, Italy; 4 Florey Institute of Neuroscience and Mental Health, University of Melbourne, Melbourne, Australia; 5 Instituto per lo Studio dei Materiali Nanostrutturati (ISMN), Centro Nazionale delle Ricerche (CNR), Via Gobetti 101, 40129 Bologna, Italy

**Keywords:** Sensors and biosensors, Polymers

## Abstract

Monitoring of bioelectric signals in peripheral sympathetic nerves of small animal models is crucial to gain understanding of how the autonomic nervous system controls specific body functions related to disease states. Advances in minimally-invasive electrodes for such recordings in chronic conditions rely on electrode materials that show low-impedance ionic/electronic interfaces and elastic mechanical properties compliant with the soft and fragile nerve strands. Here we report a highly stretchable low-impedance electrode realized by microcracked gold films as metallic conductors covered with stretchable conducting polymer composite to facilitate ion-to-electron exchange. The conducting polymer composite based on poly(3,4-ethylenedioxythiophene) polystyrene sulfonate (PEDOT:PSS) obtains its adhesive, low-impedance properties by controlling thickness, plasticizer content and deposition conditions. Atomic Force Microscopy measurements under strain show that the optimized conducting polymer coating is compliant with the micro-crack mechanics of the underlying Au-layer, necessary to absorb the tensile deformation when the electrodes are stretched. We demonstrate functionality of the stretchable electrodes by performing high quality recordings of renal sympathetic nerve activity under chronic conditions in rats.

## Introduction

Bioelectronic medicine has the vision of minimally-invasive, imperceptible interfaces that merge with neuronal tissue enabling long-term, high quality recordings and stimulation of signals from the central or peripheral nervous system^1,2^. There is evidence that the access to such signals will enable novel therapeutic means to treat currently incurable chronic or acute disease states^3,4^. To achieve this vision, material science is needed to translate current bioelectronic interfaces into a low-invasive technology in which stiff metallic electrodes are replaced by more biocompatible micro- and nanostructured electronic materials^5^. The ultimate objective is to realize electrode architectures that match the mechanical properties of tissue and show soft and stretchable properties.

In order to achieve mechanically compliant bioelectronic interfaces, several concepts and techniques from flexible and wearable electronics have been adapted to implantable devices^6–9^. Highly flexible and conformable electrode arrays for stimulation and recording have been demonstrated by employing ultrathin polymer foils as substrates onto which conductors are patterned to define electrode arrays^9,10^. The high bending flexibility allows the electrode to conformably adhere to tissue surfaces with complex morphology^11^. This was demonstrated to yield better recording signal quality and less inflammation response in comparison to stiff electrodes^12^. Further reduction in invasiveness and foreign-body response can be achieved by ultra-thin mesh shaped electrode arrays that allow interpenetration of tissue and foreign electrode material^13^. These results show that reducing device dimensions down to micro- and nanometer scales provides important means to adjust mechanical properties and related invasiveness of bioelectronic interfaces^14^. However, the materials employed are stiff metals and polymers with orders of magnitude higher elastic modulus then biological tissue^15^. The mechanical mismatch leads to interfacial stress and wear when bodily motions cause significant stretching and deformation of the interfaced nervous tissue. The possible consequences are inflammatory response and interface encapsulation or device fatigue and failure^5^. To address these problems, large research efforts are ongoing to develop implantable stretchable conductor materials that are biocompatible regarding toxicological as well as mechanical properties^16–22^. First reported stretchable bioelectronic implants^8^ employ mechanically compliant conducting micro- or nanostructures embedded on or in silicone elastomer matrix^3,23–26^ or elastic conducting polymers^27–29^. Microcracked gold thin films deposited on silicone elastomer have been used to achieve long-term chronic stimulation electrodes that maintain functionality also when implanted in a dynamic environment such as the spinal cord tissue^4^. Another recently developed implantable soft conductor is based on gold coated titanium oxide nanowires embedded in silicone matrix and was demonstrated in a stretchable electrode grid for chronic recordings from the brain surface^24^.

Crucial for bioelectronic recording and stimulation is the low-impedance conversion between ionic and electronic signals at the electrode interface. Planar metallic interfaces are limited by the relatively small specific capacitance of the ionic-double layer leading to higher thermal noise and smaller charge injection capacitance while increasing unwanted Faradaic contributions^5^. Reduction in interfacial impedance is achieved by surface treatments that increase surface roughness or that introduce surface layers with combined ionic and electronic conductivity^30,31^. In stretchable electrodes such low-impedance coatings need strong adhesion to the underlying metal and tough mechanical properties to guarantee electrode integrity also under demanding mechanical conditions. At the same time, the exposed electrode has to be directly in contact with the neuronal tissue, thus soft and elastic properties are also desired at the microscale. So far, electrodeposited Pt black and Pt-nanoparticle silicone composites have been successfully applied in chronic stretchable implants^4,24^, but such metallic nano- and microparticles have a high microscopic elastic modulus and low charge injection limits^32^. Therefore, the quest for softer, stretchable low-impedance coatings persists. Conducting polymer coatings have the potential to fulfill both requirements on low impedance as well as mechanical properties: As combined ionic and electronic conductors they offer large interfacial capacitances scaling with conducting polymer layer thickness^33^ that have already been exploited to enable high performance neuronal interfaces^34,35^. At the same time, they offer tuneable soft, almost hydrogel-like properties owing to their high ionic content leading to water uptake and swelling^36,37^. Possible drawbacks such as delamination or their rigid, brittle properties in the dry state^38^, can be addressed by adhesion promoters^39–42^ and plasticizers^37,43^ as has been demonstrated in spin-coated thin films.

Here, we report the fabrication of an elastic bioelectronic electrode that employs micro-cracked gold and an electrodeposited interface layer of the conducting polymer poly(3,4-ethylenedioxythiophene) polystyrene sulfonate (PEDOT:PSS) to combine soft micromechanics and low-impedance recording properties. We exploit Atomic Force Microscopy (AFM) performed in liquid and during stretching to show that the plasticizer polyethylene glycol (PEG) confers softer, mechanically compliant properties to the PEDOT:PSS coating that do not interfere with gold microfracture mechanics. As a consequence, low impedance electrode properties are maintained up to 40% strain. To demonstrate the functionality of our electrode we perform chronic recordings from the renal nerve in free moving rats. Small peripheral sympathetic nerves such as the renal nerve, play a pivotal role in homeostasis through its direct innervation and functional influence on a variety of organs including the vasculature, kidney, adrenal gland, heart and gastrointestinal tract. Although direct recording of symphathetic nerve activity (SNA) is difficult to achieve under chronic conditions in small animals, it is the only specific method to quantify neuronal control of specific end organs and its impact in disease states^44^. Difficulties arise due to the small and fragile structure of peripheral nerves, their embedding in dynamic tissue strongly affected by muscular motion and small signal strength due to shielding of extracellular field potentials by the epithelium layer^45^. We show that our stretchable electrode provides an optimized technology to address these difficulties and allows a simplified surgical procedure with respect to standard approaches applied in small animals^46^.

## Results

### Stretchable electrode fabrication

**Figure 1:**
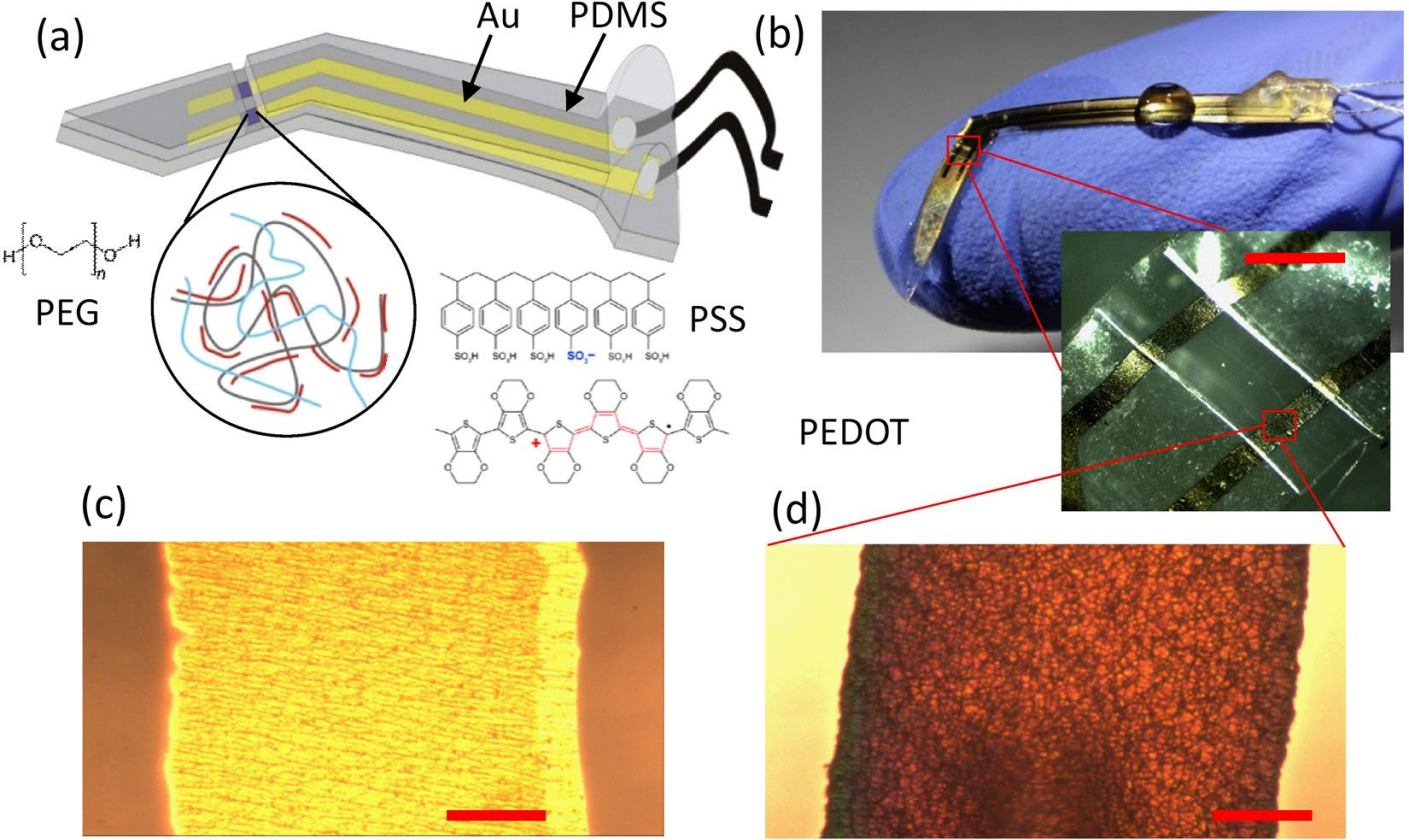
Stretchable microelectrode for small peripheral nerve recordings. (**a**) Scheme showing the electrode structure containing the materials gold, PDMS and a composite low-impedance coating of PEDOT:PSS/PEG. (**b**) Optical images featuring the electrode and the trench to contact the nerve. Scale bar 500 μm. (**c**) Optical micrograph of the gold interconnect and (**d**) the PEDOT:PSS/PEG coated electrode. Scale bar 50 μm.

To obtain the free-standing stretchable electrode as displayed in Fig. 1a we developed a simple fabrication procedure (the full fabrication flow is presented in Fig. S1, Suppl. Inf.). The substrate material is a 100 μm thick silicone elastomer layer (Polydimethylsiloxane (PDMS)). A 30 nm thick layer of microcracked gold with a 3 nm thick titanium adhesion layer is used as a stretchable conductor to define contact pads, interconnector and a pair of recording electrodes. The whole structure and its connectors to external electronics are sealed in PDMS encapsulation layer apart from a 400 μm wide opening defining a trench that exposes the two recording electrodes and serves later as a guiding structure to insert the nerve strand (Fig. 1b). In the sandwiched structure, the metallic layer on silicone maintains high conductivity under tensile strain exceeding 40% due to the presence of microcracks (Fig. 1c, Suppl. Inf. S2) that absorb the elastic deformation without breaking the interconnection between stiffer metal islands^47^. An optimized electropolymerization procedure (details below) is used to deposit conducting polymer composite thin films (Fig. 1d) that show strong adhesion to the microcracked gold film. In the final step of the fabrication, the electrode is released from the carrier substrate and a microcracked Ti/Au ground electrode is deposited on the back of the free-standing electrode to reduce the pickup of interfering EMG or ECG signals. The final shape of the electrode was optimized to facilitate the surgical procedure described later.

**Figure 2:**
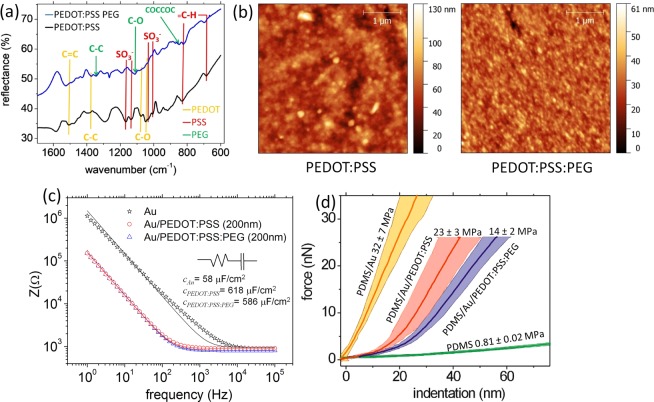
Modification of electrode surface properties with electropolymerized layer of PEDOT:PSS and PEG plasticiser. (**a**) Tip enhanced FTIR spectra of PEDOT:PSS and PEDOT:PSS/PEG coatings. (**b**) Atomic force microscopy topography maps. (**c**) Electrochemical impedance spectra. (**d**) Force as a function of AFM tip indentation depth as measured in aqueous electrolyte to extract the elastic modulus of the different material combinations.

To achieve a soft, stretchable, low-impedance electrode coating we optimized the electrodeposition of PEDOT:PSS. Electrodeposition offers the advantage to confine the deposited material on the electrodes, thus avoiding subsequent lithographic procedures. However, electrodeposited conducting polymer thin films are reported to show brittle mechanical properties and easy delamination from flat surfaces when deposited by standard amperometric procedures^38^. We improve the deposition by applying potentiometric control in inert atmosphere. In this way we avoid overoxidation and oxygen related termination of polymer formation that are attributed to lead to shorter PEDOT chains. We control the amount of deposited polymer and the coating thickness by limiting the total amount of charge deposited during the electrical procedure (see Suppl. Inf. S3). Further, we add the plasticizer polyethylene glycol (PEG) to the electropolymerization solution to tune the mechanical properties of the deposited polymer composite. PEG and other plasticizers applied in spin-coated PEDOT:PSS thin films have been demonstrated to lead to softer polymer behavior^43^. The plasticizer is incorporated into the electrodeposited film as evidenced by the tip-enhanced FTIR spectra of a composite film as shown in Fig. 2a. The appearance of a C-O stretching vibration at 1108 cm^−1^ as well as other additional adsorption bands in the C-C fingerprint region can be clearly assigned to PEG. These spectral structures remain present also after prolonged immersion of the electrode in aqueous solution (see below) demonstrating the permanent trapping of PEG chains in the composite. The surface morphology is also clearly affected by the presence of PEG. AFM investigations (Fig. 2b and Suppl. Inf. S4) of 200 nm thick films, show a reduction of surface roughness *S* and characteristic correlation length *T* (PEDOT:PSS S = 10.0 ± 0.4 nm, T = 120 ± 5 nm; PEDOT:PSS/PEG S = 4.2 ± 0.5 nm, T = 88 ± 10 nm). In PEDOT:PSS the roughness increases further in thicker electrodeposited films that show the characteristic cauliflower structures associated to electropolymerized thiophene polymers. Instead in PEDOT:PSS/PEG composites, the film thickness does not have a strong impact on characteristic morphology parameters and typical structures remain smaller, indicating an altered growth mechanism during electrodeposition.

### Electro-mechanical characterization

Crucial for the bioelectronic operation are the electrochemical impedance and the mechanical properties. Figure 2c shows the electrochemical impedance spectra for all three relevant electrode surfaces. A simple RC circuit model fits the frequency dependencies^33^. At high frequencies a flat impedance response (phase 0) is observed, corresponding to the resistance R of the electrolyte. Towards low frequencies the impedance increases following a capacitive behavior as determined by the ionic double layer at the interface. The presence of the conducting polymer composite reduces by over an order of magnitude the impedance in the capacitive regime leading to a flat frequency response in the frequency interval of interest for bioelectronic recordings (100 Hz–3 kHz). The addition of the plasticizer reduces slightly the specific capacitance per area of the layer but has no other significant effect on its ion-to-electron exchange properties.

AFM indentation experiments performed in isotonic solution reveal the micromechanical properties of the electrode as experienced by the nervous tissue. The thin layer of microcracked gold increases significantly the surface stiffness with respect to the PDMS elastomer substrate material. By approximating the force-distance curve to the Hertzian Model we compute an effective elastic modulus of E = 32 ± 7 MPa useful to compare the micromechanical properties to other surfaces. We note that the effective soft behavior results from bending of the stiff but thin gold film (E~80 GPa) under the load of the AFM tip combined with deformation of the underlying PDMS layer. In the presence of PEDOT:PSS surface coating, the effective modulus of the surface decreases. In isotonic solution we determine a value of E_PEDOT:PSS_ = 23 ± 3 MPa that compares well to the modulus of PEDOT:PSS when deposited on a stiff substrate. The presence of plasticizer reduces further the effective elastic modulus of the surface to E_PEDOT:PSS/PEG_ = 14 ± 2 MPa making it better suited to interface with soft neuronal tissue.

**Figure 3:**
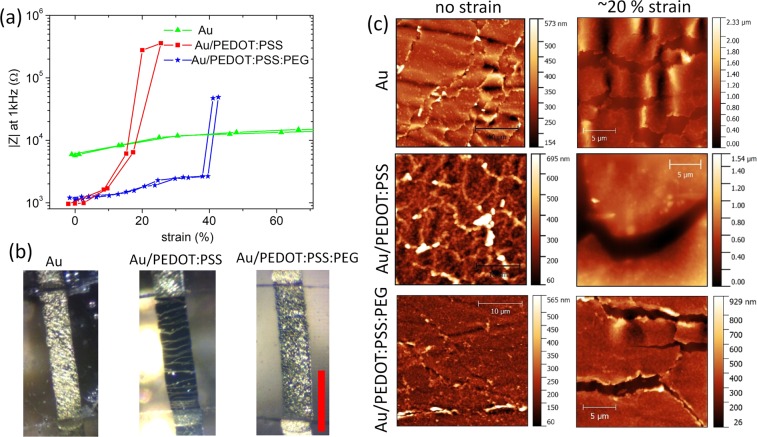
Stretchability of electrodes composed of PDMS substrate and Au, Au/PEDOT:PSS or Au/PEDOT:PSS and PEG plasticiser in phosphate buffered saline. (**a**) Electrochemical impedance magnitude at 1 kHz as a function of strain. (**b**) Optical microscopy of electrodes under 20% strain (**c**) AFM topography maps at no strain and 20% strain.

The important role of the PEG plasticizer becomes clear when the electrode properties are investigated under tensile strain. Figure 3a shows the impedance magnitude of electrodes with different coatings as a function of tensile strain. The impedance of the uncovered Au-electrode remains almost constant up to a strain value of 60%. The microcracked gold film maintains also at higher strain values a conductive percolating network that contacts the full area of the electrode thus causing no variation in its overall capacitance. We associate the small increase in impedance to single gold islands that loose contact at the borders of the electrode. This excellent stretchability of the electrode is lost in the presence of a pure PEDOT:PSS coating. Initially the impedance is much lower than pure Au, but already small strain leads to a steep increase in impedance and complete failure at strain >20%. With addition of plasticizer in the PEDOT:PSS/PEG coating we obtain an electrode that combines both, low-impedance properties with elastic stretchability. An impedance comparable to pure, unstrained PEDOT:PSS is maintained up to a strain of 40%. To investigate the reason for this behaviour we present optical and AFM images of the electrode surfaces under strain in Fig. 3b,c. Already in the optical image one can identify almost macroscopic cracks in the pure PEDOT:PSS coating that open at strain >20% The cracks are orthogonal to the strain direction and interrupt the conducting pathway along the electrode thus explaining its failure. Instead pure Au or PEDOT:PSS/PEG maintain the irregular appearance of a microcracked thin film and no long cracks are observed as a consequence of tensile strain. The AFM topographic maps further corroborate this finding. In the absence of strain, all three maps contain variations in surface height that trace the position of microcracks in the (underlying) Au film. AFM maps measured subsequently under ~20% strain then show the differences in how these surfaces absorb the tensile deformation. Comparable to findings described in literature^47,48^, the microcracks in the pure Au film open up under deformation but maintain a small extension of a few micrometers in the direction orthogonal to the strain. In this way a conducting pathway continues to connect all metallic areas on the electrode surface. In the presence of a coating on the gold layer, the pattern of the micro-cracks is strongly changed as additional forces impact on crack opening kinetics. The effect is most drastic in the presence of pure PEDOT:PSS. Almost no opening of microcracks is observed under strain but instead the strain is localized in fewer cracks that accordingly show much wider openings causing complete loss of conductivity in the layer. These cracks are combined also with a significant reduction in surface height, exceeding the pure thickness of the PEDOT:PSS coating (200 nm) due to the necessity of the elongated elastic substrate to maintain constant volume. Instead if the plasticizer is present, microcrack opening is again observed during application of tensile strain and the overall surface topography resembles the pure microcracked gold thin film. Only smaller differences such as a reduced surface undulation and straighter crack borders can be associated to the presence of the polymer coating. Therefore, up to 40% strain the microcracks in Au/PEDOT:PSS/PEG can contain the strain similar to the pure gold film without giving rise to a macroscopic crack that blocks conductivity.

**Figure 4:**
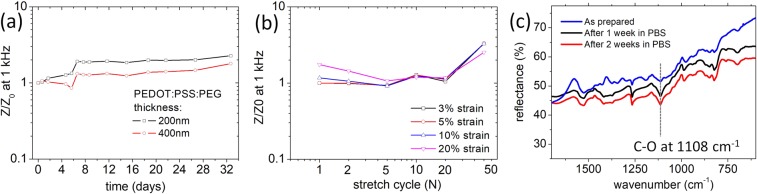
Stability of PEDOT:PSS/PEG based low-impedance electrodes. (**a**) Normalized impedance at 1 kHz as a function of immersion time in 38 °C isotonic PBS solution. (**b**) Normalized impedance at 3%, 5%, 10% and 20% tensile strain as a function of number of repetitive stretching cycles with maximum strain of 40% performed while immersed in PBS. (**c**) Tip enhanced FTIR spectra obtained on the electrode after different times of immersion in PBS showing the chemical stability of the coating. The intense C-O stretching band of PEG at 1108 cm^−1^ is clearly visible after soaking the electrodes in PBS for two weeks.

In Fig. 4 we present data regarding the long-term stability and fatigue behaviour of the PEDOT:PSS/PEG coated stretchable electrodes. Variations in the electrode impedance at a frequency of 1 kHz as characteristic for bioelectronic signals are investigated. Figure 4a shows how the impedance evolves with the time of immersion in 38 °C isotonic solution that mimics degradation in the body environment. Independent of thickness, the films show good stability and low impedance properties are maintained over several weeks. Also repetitive stretching cycles as shown in Fig. 4b do not lead to a significant degradation. Only when 40% stretching cycles exceed 50 repetitions, accumulation of defects at the border of the electrode is observed that causes an increase in the series resistance but do not affect the films volumetric capacitance. Long-term immersion in water does also not alter the chemical composition of the coating as demonstrated in Fig. 4c. PEG molecules do not leak out into the solvent but remain entrapped in the film as demonstrated by the strong C-O stretching band uniquely attributed to PEG that remains present also after two weeks of immersion in PBS.

In summary, the experiments show that PEG plasticiser enables to achieve a low-impedance PEDOT:PSS polymer coating that maintains the reversible stretchability of the gold micro-cracked electrode. We suggest that the strong hydrophilic properties of PEG increase the water content in the film and as a consequence a higher polymer chain mobility and smaller crystalline PEDOT rich regions appear in the conducting polymer nano-composite causing the changes in material properties. The improved coating does not interfere with the gold microcrack network mechanics that allow the film to elongate without creating macroscopic cracks and interruption of electrical conductivity.

### Chronic recordings from sympathetic nerve

**Figure 5:**
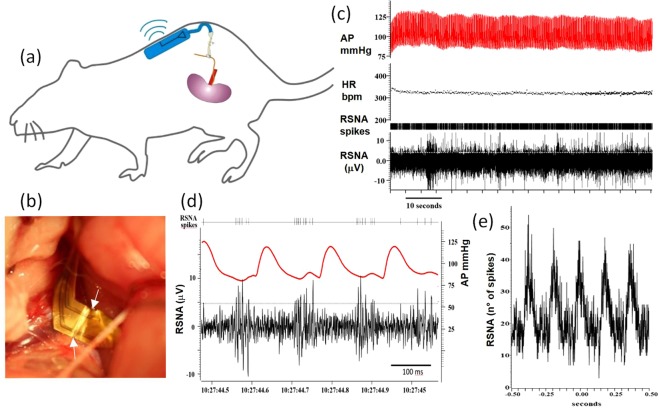
Chronic recording of rat sympathetic nerve activity (RSNA): Scheme showing the elastic electrode connected to the renal nerve. The amplification and transmission electronics is located on the back under the skin. (**b**) Optical micrograph of electrode positioned below renal nerve before sealing with silicone elastomer. (**c**) trace of recorded signals with radiotelemetry: Renal sympathetic nerve activity (RSNA), aterial preassure (AP), heart rate (HR). (**d**) detail of RSNA (black) and AP (red) trace. (**e**) crosscorrelation of RSNA spiking activity and AP.

To demonstrate the facile handling of the elastic electrode and its optimized soft mechanical and electrical low-impedance properties we performed recordings under acute and chronic conditions from the renal nerve of Sprague-Dawley rats. Figure 5a shows all components of the neural recording assembly comprising the elastic electrode and a wireless recording board that is small enough to be placed subcutaneously in the animal. Before insertion of the nerve interface, animals were equipped with a telemetric arterial blood pressure monitor. Then the abdomen was opened and a portion of the target nerve was dissected away from all connective tissue. The opening trench of the stretchable electrode was placed with tweezers below the nerve strand (Fig. 5b). Here the asymmetric shape of the electrode was optimized to allow facile insertion into the surgical opening and manipulation of the electrode with tweezers. An electric contact was established by inserting the nerve strand into the trench. All of the fluid around the nerve and electrode was removed with small absorbent spears. In this intraoperative situation, acute recordings were performed by activating the wireless recording board and transmitting the filtered and digitized signals to the external data acquisition computer. To further prepare the interface for chronic recordings we used a drop of a 2-component silicone elastomer (Kwik-Sil) to seal the nerve strand in the trench of the elastic electrode. The elastic interconnector was then sutured to neighbouring muscular tissue to keep the electrode in position during the closure of the abdominal muscle with discontinuous absorbable sutures. The connected wireless recording board was placed subcutaneously on the back of the rat and finally the skin was sutured closed (see Suppl. Inf. S5 for further information regarding the surgical procedure**)**.

After recovery from the surgical procedure the animals showed normal behaviour with usual food uptake and unrestrained motional activity. No signs of pain or distress or other significant impairment due to the implanted recording system were observed. In chronic recording experiments the wireless recording board was activated by positioning a magnet close to the animal. We analysed only signal traces that were recorded while the animal was awake but in a resting position. Figure 5c shows a data trace that was acquired with telemetry at day 14 after surgery. Over the shown recording period of 80 s a constant physiological arterial blood pressure (AP) 85–120 mmHg and heartrate (HR) of 330 bpm were observed while the RSNA signal shows spiking activity. The same signals plotted on a shorter time scale (Fig. 5d) reveal that the spiking activity is synchronized to the blood pressure signal. Several sympathetic bursts of up to 10 μV related to compound action potentials are observed after each peak in systolic blood pressure. Instead during pressure maxima only smaller random voltage fluctuations of 1.3 μVrms are present that we associate to thermal amplifier noise. No signs of interfering ECG or EMG signals are present due to the effective shielding of the ground electrode on the back side of the elastic electrode. The rhythmicity of the RSNA signal with blood pressure is confirmed also over longer recording periods. Figure 5e shows the correlation between the time lag to the maxima in blood pressure and the number of RSNA spikes obtained for a recording trace of 80 s. Maxima in activity clearly appear as multiples of the heart beat interval of 180 ms. The observed pulse-synchronous activity is typical for sympathetic nerves such as the renal, splanchnic or lumbar nerve and is usually used in recordings under acute conditions to verify the recording quality. The ability to capture these oscillations in chronic experiments highlights the quality of our stretchable, low impedance interface. In comparison to traditional peripheral nerve recordings achieved in small animals with microwires^46,49^ we see two main advantages of our approach: First, the compact design and the possibility to easily adapt the shape facilitate the surgical procedure alleviating the need to position and suture individual microwires. Second, we suggest that the stretchable properties of the electrode and its interconnector are important to achieve longterm recordings. The renal nerve is embedded in muscular tissue and during animal activity the area is subjected to motion and displacements. In traditional peripheral nerve interfaces in small animals made with stiff microwires, animal motions are related to device failure in chronic conditions occuring a few days after surgery^46,49^. Instead our fully elastic electrode can adapt to the dynamic environment without generating interfacial forces that potentially damage or displace the device.

## Conclusions

In conclusion, our work demonstrates a facile fabrication route to obtain low-impedance stretchable electrodes for bioelectronic interfaces based on microcracked gold deposited on silicone elastomer. A low impedance interface to the bioelectronic signals is achieved by electrodeposition of the conducting polymer PEDOT:PSS. In contrast to other deposition techniques such as spin-coating^10,43^ or spray-coating^50^, electrodeposition makes an additional lithography step to pattern PEDOT:PSS unnecessary, as the layer is confined to the electrode with electrically controlled thickness. Importantly, we modify the electrodeposition technique to include the plasticiser PEG in the deposited conducting polymer layer. In this way we alter the mechanical properties of the PEDOT:PSS to obtain a stable low impedance coating that is compatible with the micro-fracture mechanics of the underlying gold film necessary to maintain electrical conductivity during tensile strain. Identical electrodes fabricated with pure PEDOT:PSS films deposited according to standard procedures lead to macroscopic crack formation and electrode failure. Microscopic investigations under strain show that the improved properties of the PEDOT:PSS/PEG coating are associated to an altered growth mechanism that changes the thin film morphology achieving a softer elastic behaviour and lower Youngs modulus. To demonstrate the unique possibilities opened by our fabrication technique, we developed a bipolar microelectrode connected to a wireless amplifier board to interface the renal nerve in free moving rats. Bioelectronic recording experiments performed under chronic conditions show that the elastic and low-impedance properties of the microelectrode enable stable recording of sympathetic nerve activity for several days to weeks in unconstrained small laboratory animals. The presented electrodes simplify the surgical procedure and reduce the risk of mechanical failure in the dynamic environment of peripheral nerves embedded in muscular tissue. The access to sympathetic nerve signals under chronic conditions is relevant to understand their role in different chronic or acute disease states in basic animal models.

## Methods

### Elastic electrode fabrication

The fabrication process is reported in Fig. S1. Glass substrates (75 × 25 mm^2^) were cleaned by sonication in distilled water/acetone/isopropanol baths, 15 minutes for each step, then dried with nitrogen flux. Afterwards, substrates were treated with oxygen plasma (100 Watt for 5 minutes) and a sacrificial layer of Polyacrylic acid (PAA) dissolved in DI water (10% in volume) was spin coated (500 RPM for 20 s), with subsequent annealing at 80° for 3 min. Polydimethylsiloxane (PDMS) prepolymer and Curing Agent (Sylgard 184, Silicone Elastomer, Dow Corning) were mixed in 10:1 weight ratio and spin coated on top of PAA layer (300 RPM for 3 min) followed by annealing at 90° for 1 h. A similar method was used for Polyethylene terephthalate (PET) substrates, that were used for the upper PDMS layer for the encapsulation of the Au interconnects. Then, 3 nm of titanium (Ti) and 30 nm of gold (Au) were deposited on glass/PAA/PDMS substrates by laser electron beam evaporation, with the pattern visible in Figs S1–2. To seal the interconnects glass/PAA/PDMS and PET/PAA/PDMS substrates were plasma activated in air (100 Watt, 30 s) and stuck together, after a proper trench was realized to uncover the microelectrodes (Figs S1–3). Electrical connections were then made, using silver paste to connect the wire with the Au pad, sealing and fixing it with Silicone Dow Corning 734 Clear. PEDOT:PSS or PEDOT:PSS/PEG were then electrochemically deposited on uncovered Au microelectrodes, using chronoamperometric method (E = 1.2 V, Metrohm PGSTAT 204) using saturated calomel electrode (SCE) as reference and a Pt counter electrode. The electropolymerization was carried out in a deaerated solution of distilled water containing 10 mM EDOT, 0.1 mM PSS, and 5 mM PEG under a constant magnetic stirring. Finally, the electrode was trimmed and released from the carrier substrate and a microcracked Ti/Au ground electrode is deposited on the back of the free-standing electrode, then connected with a wire and silver paste, as described above. For wireless *in-vivo* recordings the two electrode wires and the ground wire were connected to a battery operated differential amplifier and transmitter (Data Science International).

### Characterization techniques

Attenuated total reflection FTIR measurements were performed on a PerkinElmer FT-IR spectrometer Spectrum Two. Electrochemical impedance spectra were acquired with a Metrohm PGSTAT 204 in PBS 0.1 M. Strain was applied by a tensile tester immersed in the solution. A Park NX10 system was used for Atomic Force Microscopy. Non-contact topographies were acquired with a PPP-NCHR tip (Nanosensors). Indentation experiments were performed with a NSC36_B (k = 2 nN/nm) in 0.1 M PBS solution. The Hertizian model for a parabolic tip shape was used to fit the indentation curves and to provide the effective elastic modulus. Topographies during strain were obtained by clamping the sample in a tensile tester mounted on the AFM sample holder^51^.

### *In-vivo* renal sympathetic nerve recordings in the rat

The protocol was approved by the Florey Institute of Neuroscience and Mental Health Animal Ethics Committee, under the guidelines set out by the National Health and Medical Research Council of Australia “Code of Practice for the Care and Use of Animals for Experimental Purposes in Australia”.

Sprague-Dawley rats (350–450 g) were anesthetized with 2% isoflurane, and their body temperature was kept at 37.0 ± 0.5 °C with a thermal blanket. The left femoral artery was carefully dissected from the vein and nerve, and telemetry device for measuring blood pressure implanted. Arterial blood pressure (AP) was measured with PA-C40 telemetric transmitters (Data Sciences International, MN). The animal was then placed in the prone position, a left longitudinal incision made on the left flank and the soft tissue was dissected exposing the retroperitoneal cavity. The renal nerve was carefully dissected from the perineural connective tissue and positioned on the electrodes (Fig. 1a), which were stabilized with Kwik-Sil (World Precision Instruments, FL). The wires were stitched onto the back muscles and the retroperitoneal cavity was closed. The transmitter for nerve impulses (Data Sciences International, MN) was secured subcutaneously between the scapulae.

The animals were allowed to recover for a week prior to recording of physiological signals. Renal sympathetic nerve activity (RSNA) was recorded with the 50–1000 Hz band and sampled at 10000 Hz. The AP and RSNA telemetric signals were then digitized using a Power1401 (Cambridge Electronic Design, Cambridge UK), using Spike2 v7.02 software. Number of spikes was determined by quantifying the spikes that overcame the threshold set above the noise level (Fig. 4d).

The datasets generated and analysed during the current study are available from the corresponding author on reasonable request.

## Supplementary information

Supplementary Information
