# Supplementary material for: Stretchable Low Impedance Electrodes for Bioelectronic Recording from Small Peripheral Nerves
Source: Sci Rep. 2019 Jul 22;9:10598. doi: 10.1038/s41598-019-46967-2 (PMC6646361; doi:10.1038/s41598-019-46967-2)
Supplement: Supplementary file 1 — Supplementary Information [file 41598_2019_46967_MOESM1_ESM.pdf]

Supplementary Information

Stretchable Low Impedance Electrodes for Bioelectronic Recording from Small Peripheral Nerves

Francesco Decataldo, Tobias.Cramer, Davide Martelli, Isacco Gualandi, Willian S. Korim, Song T. Yao, Marta Tessarolo, Mauro Murgia, Erika Scavetta, Roberto Amici, Beatrice Fraboni*

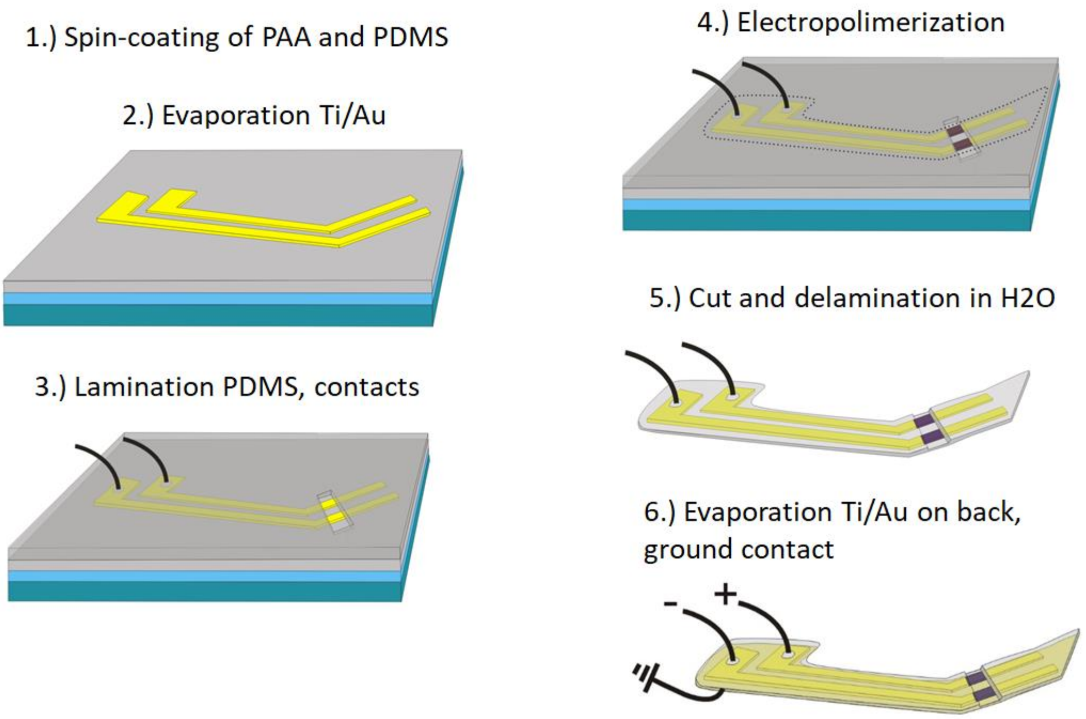

Figure S1. Fabrication of elastic low-impedance microelectrodes. The procedure involves 6 different fabrication steps.

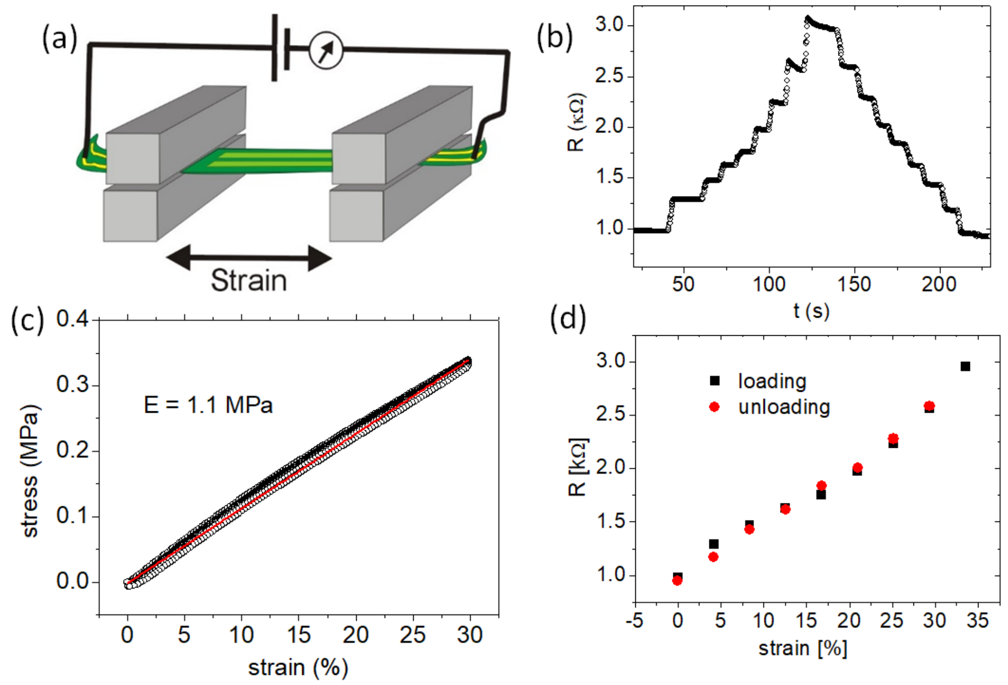

Figure S2. Electromechanical characterization of electrode interconnects: (a) scheme of the experiment. (b)interconnect resistance R as a function of time while increasing and then decreasing tensile strain was applied. (c) Tensile stress of interconnect as a function of strain. From the slope an elastic modulus of 1.1 MPa is obtained. (d) Interconnect resistance as a function of strain.

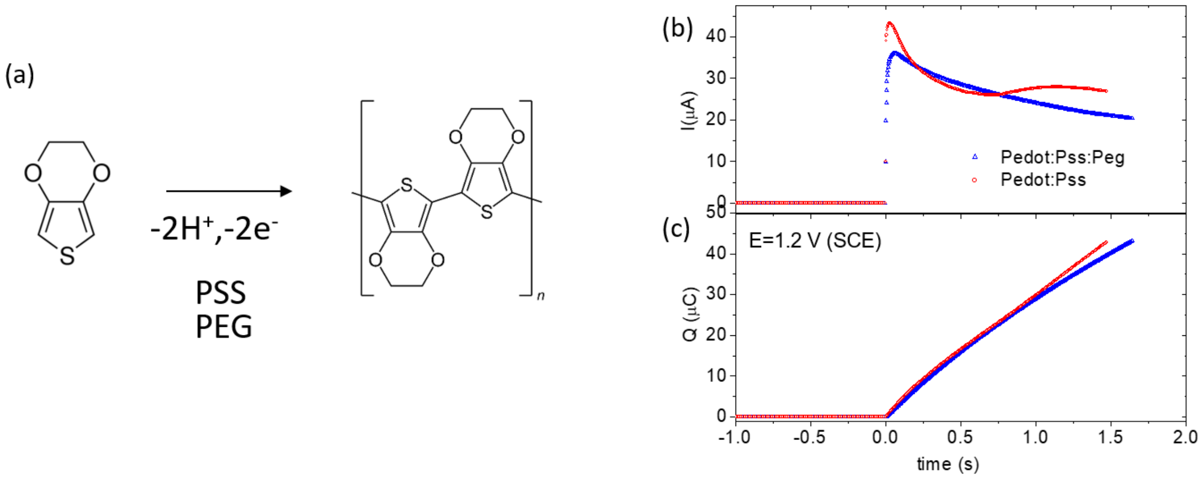

Figure S3. Electrodeposition of semiconducting polymer composite. a) Chemical reaction scheme; (b) Electrochemical current I obtained during chronoamperometric deposition; at t=0 a voltage of E(vs SCE)=1.2 V is applied. (c) The deposited charge as measured during the deposition. Once the desired amount is deposited, the potential will be set back to ground and the deposition is stopped.

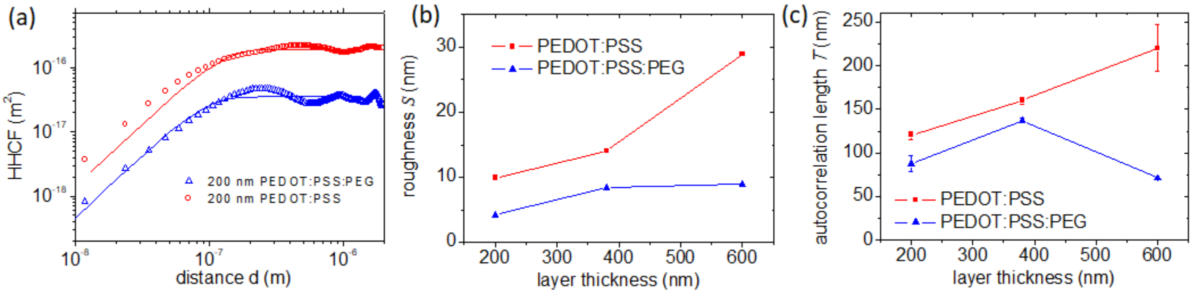

Figure S4. Quantitative analysis of AFM topography of 200 nm PEDOT:PSS and PEDOT:PSS+PEG thin films. (a) The Height-Height Correlation Function (HHCF) is calculated. A fit to the function $HHCF = 2S^2\left[1-\exp\left(-d^2/T^2\right)\right]$ is performed. The parameters S and T represent the sample roughness and the characteristic autocorrelation length respectively. (b,c) S and T are plotted as a function of the thickness of the deposited semiconducting polymer composite.

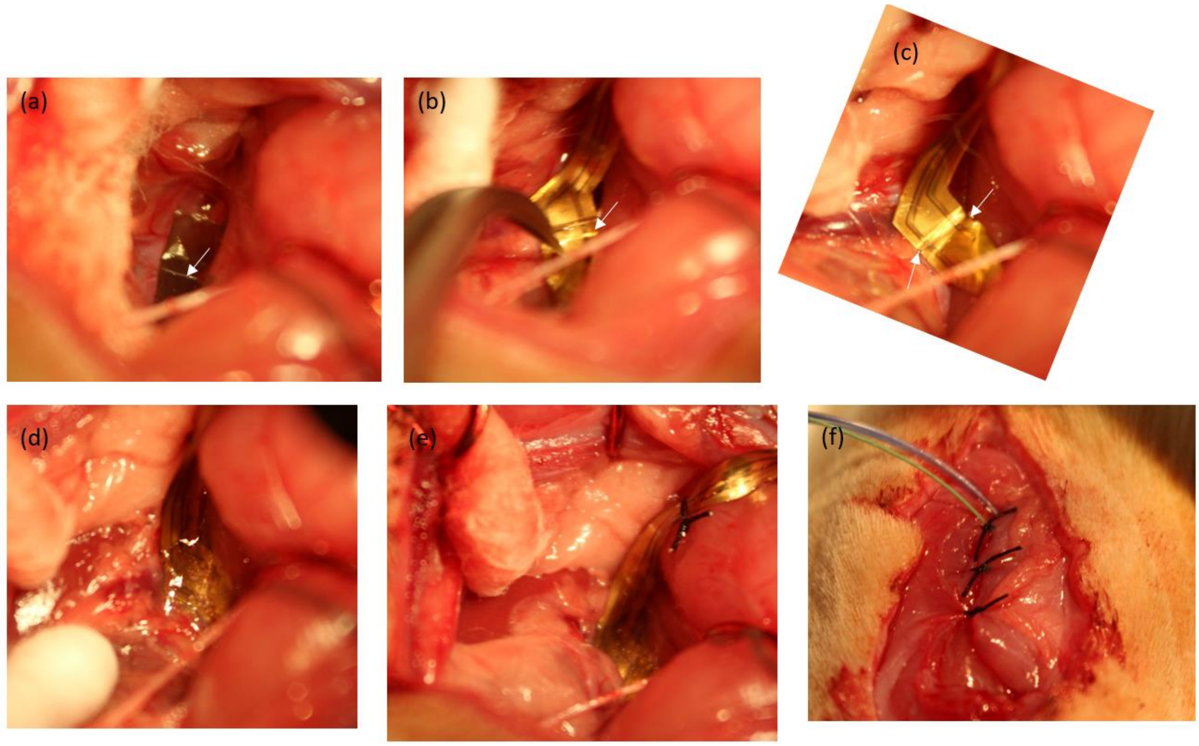

Figure S5. Optical micrographs taken during implantation of the elastic microelectrode on the renal nerve. (a) The renal nerve is isolated. (b) the microelectrode is pulled below the nerve. (c) the nerve is inserted into the trench of the electrode. (d) the trench with the nerve is sealed with selfcuring silicone. (d) The interconnect is sutered on the xxx muscle. (e) the tissue is closed.
